# Impact of disease on diversity and productivity of plant populations

**DOI:** 10.1111/1365-2435.12552

**Published:** 2015-09-23

**Authors:** Henry E. Creissen, Tove H. Jorgensen, James K. M. Brown

**Affiliations:** ^1^ Crop Genetics DepartmentJohn Innes CentreNorwich Research ParkNorwichNR4 7UHUK; ^2^ School of Biological SciencesUniversity of East AngliaNorwichNR4 7TJUK; ^3^ Department of BioscienceAarhus University8000Aarhus CDenmark; ^4^ Present address: Department of Crop Science, TEAGASC CropsEnvironment and Land Use Programme, Oak ParkCarlowIreland

**Keywords:** *Arabidopsis thaliana*, compensatory interactions, genotype mixtures, *Hyaloperonospora arabidopsidis*, overyielding, plant disease, *Turnip yellows virus*, yield stability

## Abstract

1. Experiments were conducted on the role of intra‐ and inter‐genotypic competition in ecological processes operating at the population scale in diseased plant populations.
2. Combinations of *Arabidopsis thaliana* genotypes showing variation for phenotypic traits relating to competitive ability and pathogen compatibility were infected with the oomycete *Hyaloperonospora arabidopsidis* and *Turnip yellows virus* in separate experiments. Plant fitness and competitive ability were estimated from phenotypic measurements.
3. Pathogen‐induced reduction in competitive ability for susceptible genotypes increased the competitive ability of resistant genotypes, resulting in maintenance of yield via competitive release. The two diseases had different effects on competitive interactions between plants. In experiments involving the oomycete, the highest yields were produced by mixtures of two weakly competing genotypes.
4. The *Arabidopsis* model system has elucidated the ecological processes by which compensatory competitive interactions can increase the buffering capacity of plant populations under pathogen attack. Highly competitive genotypes may not maximize the productivity of the population as a whole, as they may over‐yield at the expense of less competitive, more productive genotypes. The specific outcomes of competitive interactions cannot be generalized because they depend on the disease and the host genotypes.

## Introduction

The relationship between plant diversity, ecological stability and ecosystem productivity is of great importance to natural systems. Plant pathogens alter such relationships by affecting plant fitness, reducing the growth and competitive ability of diseased plants which can impact heavily upon plant population and community structure (Burdon, Thrall & Ericson 2006; Bradley, Gilbert & Martiny 2008; Maron *et al*. 2011; Latz *et al*. 2012). Pathogens can promote plant biodiversity by preventing competitive exclusion if they have a greater negative impact upon the dominant species in a community, such that a trade‐off exists between plant competitive ability and susceptibility to pathogens (Alexander & Holt 1998; Bradley, Gilbert & Martiny 2008; Allan, van Ruijven & Crawley 2010). However, if pathogens have a greater detrimental impact upon uncommon and less competitive species then biodiversity will be reduced (Peters & Shaw 1996). Studies investigating the effect of biodiversity on the system's ability to buffer against disease have been largely observational; for example, increased species richness reduces disease caused by foliar and soil borne pathogens and increases productivity in grassland communities (Allan, van Ruijven & Crawley 2010; Maron *et al*. 2011). Despite the pertinence of understanding the impact of pathogens on plant diversity at all levels of biological organization, studies of natural systems are rare and there has been a lack of experimental tests of mechanisms of pathogen‐induced changes to diversity. Theory predicts that increased stability can be achieved through resistance to change or by recovery after perturbation (Pimm 1984).

Host fitness and competitive ability can be reduced by susceptibility to pests or pathogens, or through costs of defence (Damgaard & Jensen 2002; Tian *et al*. 2003; Bedhomme *et al*. 2005; Brown & Rant 2013). When costs of resistance are associated with reduced plant growth or reproduction, they may be traded‐off against competitive ability since the latter may be associated with increased allocation to growth (Chase *et al*. 2002; Viola, Mordecai & Jaramillo 2010). Investment in vegetative growth can increase tolerance to indirect costs of infection, increasing competitive ability through greater plant fitness (Pagan, Alonso‐Blanco & Garcia‐Arenal 2009). However, costs of defence can decrease or even disappear in the presence of inter‐plant competition because of the reduced fitness and competitive ability of more heavily diseased, susceptible plants, as in *Arabis perennans* infected by *Plutella xylostella* (Siemens *et al*. 2003). These findings raise questions about the ambiguous role of defence costs in plant ecology.

Experiments in which diversity and environmental stresses are manipulated can reveal the mechanisms of increased productivity and ecological stability in diseased populations and provide insight into the relative costs and benefits of plant defence in plant communities. *Arabidopsis thaliana* (Brassicaceae) has traits, including small size and short generation time, which make it particularly suitable as a model system for experiments on competition under glasshouse conditions in which environmental conditions can be controlled. This study examined the effect of plant genotypic diversity in stabilizing plant productivity in populations under pathogen attack. It experimentally tested the hypotheses that compensatory interactions, in which declines in productivity of some genotypes are compensated by increases in others, increases yield stability in mixtures containing the most phenotypically diverse genotypes, and that this effect is greatest when susceptible and resistant genotypes are combined in the presence of the pathogen. The general applicability of these hypotheses was tested using two very different parasites, the oomycete *Hyaloperonospora arabidopsidis* and *Turnip yellows virus*. We predicted that dissimilar pathogens would have different effects on the relationship between plant diversity, stability and productivity because of differences in disease transmission, progression and impact on plant fitness.

## Materials and methods

### The Model System

*Arabidopsis thaliana* is a useful model for studying the effect of competition on productivity because seed number correlates positively with vegetative biomass (Aarssen & Clauss 1992; Clauss & Aarssen 1994). To study the ability of *A. thaliana* genotype mixtures to buffer against disease and stabilize yield in different environments, two pathogens were selected that differ greatly in their taxonomy, transmission and impact on plant fitness.

*Hyaloperonospora arabidopsidis* (*Hpa*) is an obligate oomycete pathogen causing downy mildew in natural populations of *A. thaliana* (Koch & Slusarenko 1990; Holub, Beynon & Crute 1994). *Arabidopsis thaliana* genotypes vary greatly in their interactions with *Hpa* isolates in terms of resistance (Nemri *et al*. 2010) and tolerance (Salvaudon, Heraudet & Shykoff 2008). Moreover, plant competitive ability can be altered by the presence of this pathogen (Damgaard & Jensen 2002). This pathosystem is therefore suitable to study mechanisms of increased yield and yield stability in plant genotypic mixtures.

The polerovirus *Turnip yellows virus* (TuYV) is a major pathogen of oilseed rape (*Brassica napus*) with potential to decrease yield by 26% (Jay, Rossal & Smith 1999; Stevens, McGrann & Clark 2008). TuYV is insect‐borne with the main vector being the peach–potato aphid (*Myzus persicae*). No known *A. thaliana* genotypes are resistant to TuYV, but there is variation in tolerance to the virus (Stevens *et al*. 2005). This virus was used as it represents a group of agriculturally important pathogens of which some can infect *A. thaliana*. Viruses and oomycetes use host resources in very different ways. Experiments with TuYV can indicate how universal the consequences of pathogen infection for plant fitness and competitive ability are.

### Experimental Design

Four *A. thaliana* genotypes were selected for the *Hpa* experiment and two for the TuYV experiment (see below). Plants were sown in pots (70 × 70 × 70 mm) with four plants per pot 30 mm apart, generating intense competition (Creissen, Jorgensen & Brown 2013). Plant height, rosette size and flowering time were measured for two focal plants in each pot. Limited space prevented bagging of the remaining two plants so they acted as competing neighbours only. Plants were cultivated as monocultures and as mixtures of two or four genotypes to assess competition between different genotypes.

### Plant Growth Conditions

Seeds were sown in 8:1 compost: grit (compost: Levington F2 soil, N:P:K 150:200:200 mg L^−1^, pH 5·3–5·7) and incubated at 4 °C for 4 days to break dormancy. Seedlings were then moved to a glasshouse to germinate at 18/12 °C 8/16 h day/night supplemented with high‐pressure sodium lighting (240 μmol m^−2^ s^−1^). Ten days later, they were transplanted into the experimental design. When plants began to flower (phase 6, Boyes *et al*. 2001), glasshouse temperatures were increased to 23/16 °C day/night to accelerate maturation.

### Experiments with *Hyaloperonospora arabidopsidis*

Four genotypes of *A. thaliana* (Van‐0, Ga‐0, NFA‐10, NFA‐8) were selected from an initial screen of 15 genotypes, based on phenotypic variation in traits related to fitness, including rosette size and seed production, and compatibility with *Hpa* in the absence of competition (Table S1a, Supporting information). The proportion of resistant to susceptible genotypes selected for this experiment (50:50) is typical of natural populations of *A. thaliana* plants (Rose *et al*. 2004). Variation in flowering time was restricted to a window of 1 week so the peak requirement for resources would occur at a similar time. *Hpa* isolate Emoy2 was maintained on a susceptible host genotype, NFA‐8, and inoculated by spraying a suspension of 5 × 10^4^ conidia mL^−1^ in distilled water onto 18‐day‐old plants (Reignault *et al*. 1996). After inoculation, plants were covered with a transparent plastic lid to maintain humidity (90–100%). Control plants were sprayed with water and subjected to the same conditions. Control and inoculated plants were grown in adjacent compartments of the same glasshouse to make growing conditions similar as possible. Marginal differences in temperature and humidity were observed between rooms in the same experimental repeat (data not shown) so rooms were swapped between repeats in a split‐plot crossover design. This experimental design was chosen over the alternative of conducting the experiment in the same room and spraying control plants with fungicide because of the effects of such chemicals on plant physiology.

Each experiment included 22 treatments, namely the four genotypic monocultures, all six possible two‐genotype combinations (two‐way mixtures) and a mixture of all four genotypes (four‐way mixture), all in the presence and absence of the pathogen. In each experiment, there were 20 pots of each of the 11 monocultures and mixtures within each pathogen treatment and the 220 pots within each glasshouse were completely randomized. Two independent experiments were carried out beginning in October 2011 and March 2012. During the second experiment, glasshouse temperatures were more variable (standard deviation [SD] of mean temperature in 2011 was 5·4 °C compared to 6·2 °C in 2012) and the maximum temperature was higher (2011: 32 °C, 2012: 38 °C) as was humidity (2011: 56%, 2012: 63%). This resulted from a few days of strong sunshine during March and April 2012.

Measurements on focal plants to assess fitness included days to first flower (phase 6, Boyes *et al*. 2001), rosette diameter after 5 weeks growth and total seed mass. Disease severity was assessed twice. At 6 days post‐inoculation (dpi), the number of leaves bearing conidia and the proportion of leaves infected were recorded. At 10 dpi, plants were scored using the following 0–4 scale of disease development:

0 = no signs of sporulation.
1 = a few sporulating conidia detectable using a hand lens   (4× magnification).
2 = 1–33% leaf area diseased.
3 = 34–66% leaf area diseased.
4 = 67–100% leaf area diseased.

Plants were bagged with individual clear, micro‐perforated bags when the first siliques began to ripen to ensure all seeds were collected. The natural logarithm of response ratios (lnRR; Cahill 1999) were calculated for each genotype to assess mixture performance and competitive ability in the presence and absence of the pathogen.

### Experiments with *Turnip Yellows Virus*

A preliminary screen of 12 genotypes revealed only two genotypes (Col‐0 and Ler‐1) that differed significantly in tolerance to TuYV using the same criteria for phenotypic variation as for *Hpa* infection (Table S1b). Tolerance describes the ability of a plant to prevent itself from being damaged even though it is infected by a parasite (Brown & Handley 2005). It was characterized in this study by reductions in fitness (seed production, rosette size) in the presence of high levels of viral antigen within infected leaf tissue, assessed by enzyme‐linked immunosorbent assay (ELISA) (Clark & Adams 1977) 4 weeks after inoculation. Col‐0 suffered greater yield loss than Ler‐1 despite similar viral titres within leaf tissue 4 weeks after infection with TuYV (data not shown). Ler‐1 was therefore more tolerant to TuYV than Col‐0. No significant effect of non‐viruliferous aphids on plant fitness was found (data not shown).

After 14 days in the glasshouse, plants were inoculated with TuYV isolate BrYV‐GB by placing three viruliferous *M. persicae* (RRes genotype 0; Bos *et al*. 2010) aphids onto each plant using a paint brush. All trays of inoculated plants were covered with clear plastic lids to prevent the spread of aphids onto uninoculated plants. The experiment, beginning in October 2012, included six treatments, namely the two genotypic monocultures and a mixture of both genotypes, both in the presence and absence of the pathogen. There were 25 replicates of each monoculture and 50 of the mixture within each pathogen treatment, and the 200 pots were completely randomized. The planting design was the same as in the *Hpa* experiment except for the absence of a four‐way mixture. All pots received a compost drench with the insecticide Intercept^™^ 70 WG (Scotts UK, active ingredient imidacloprid, 0·2 g L^−1^ water) 1 week after inoculation to kill aphids and thus prevent further virus transmission. After 7 days, once all aphids were dead, the plastic lids were removed. Virus‐inoculated and control plants were grown in the same glasshouse compartment at 20/18 °C 16/8 h day/night. Forty plants of each genotype per treatment (aphids/no aphids) grown among the focal plants in separate pots were tested by ELISA to confirm the presence of TuYV in inoculated plants and its absence in control plants. Days to first flower, rosette diameter and total seed mass were measured.

### Statistical Analysis

Linear mixed modelling was used to evaluate differences in seed mass, rosette size, flowering time and disease between monocultures and mixtures of *A. thaliana* genotypes. The model included the main effect of each factor and all interactions between them. Fixed factors included genotype, presence/absence of the pathogen and cultivation (monoculture, two‐way or four‐way mixture). A separate linear mixed model analysed the effect of genotype and cultivation on each trait measured. All nonsignificant (*P *>* *0·05, *F*‐test) interaction terms were removed from the analysis and the model rerun. The random effect for each model was the pot in which the plants were grown. All statistical analysis was conducted using genstat v.14 (VSN International 2011).

lnRR was used to measure competitive intensity of focal plants, calculated as the natural logarithm of yield in the mixture divided by yield in monoculture. A negative lnRR indicates that reproductive output was reduced by the effect of competition while a positive lnRR indicates that a plant over‐yielded in the presence of its neighbours.

Covariances in seed production were used to estimate compensatory dynamics in the pairwise interaction experiments (two‐way mixtures). Negative covariances indicate compensatory dynamics, and positive covariances indicate correlated dynamics (Gonzalez & Loreau 2009).

Dynamic stability (Type 2; Lin, Binns & Leftovich 1986; Becker & Léon 1988) of seed production for each planting treatment (monocultures, the two‐way mixtures and the four‐way mixtures) was assessed by SD of the logarithm of seed mass. A lower SD indicates less genotype‐by‐environment interaction (Lin, Binns & Leftovich 1986; Becker & Léon 1988). The effects of experiment, disease treatment and their interaction were removed by fitting those factors to log (seed mass) in a multiple regression model and SD of residuals from that model was used as a measure of stability for the three planting treatments. Note that, as SD is not biased by sample size, this method does not require equal replication of treatments, unlike other measures of stability. SD was compared by *F*‐tests of their squared values (i.e. variance ratio).

## Results

### *Hyaloperonospora arabidopsidis*

**Figure 1:**
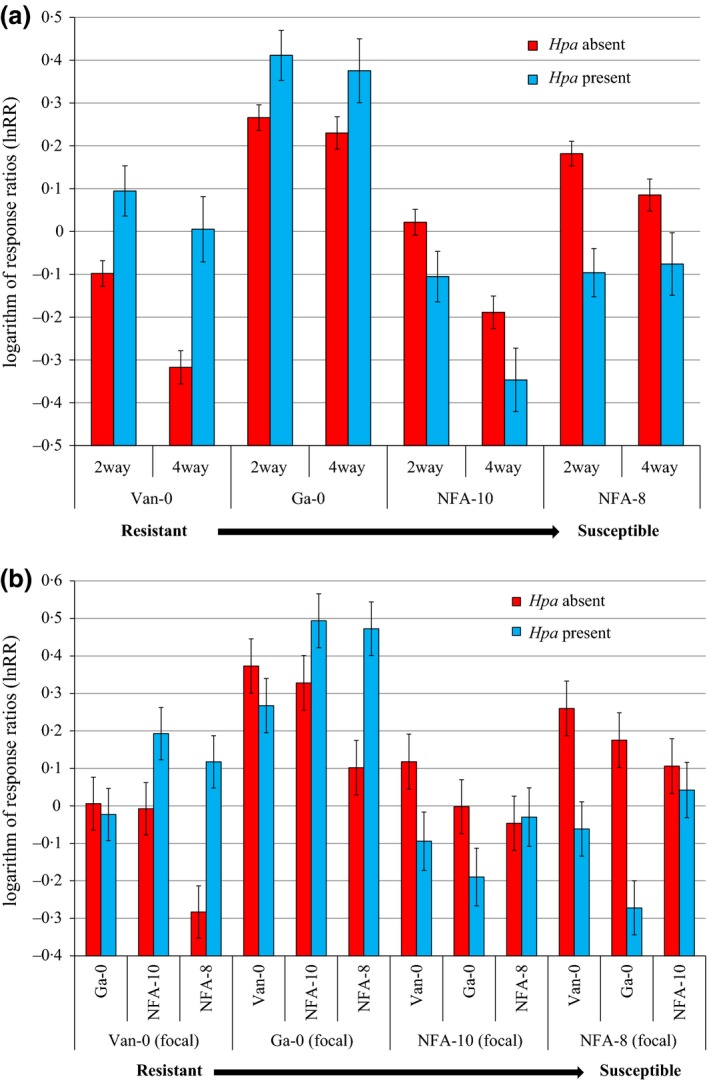
(a) Competitive intensity measured by the logarithm of response ratios (lnRR) (log seed in mixture/seed in monoculture) of four *Arabidopsis thaliana* genotypes in the presence and absence of *Hyaloperonospora arabidopsidis* (*Hpa*) in two‐way and four‐way mixtures of genotypes. Negative lnRR indicates a reduction of yield through competition while positive lnRR indicates increased yield in the presence of other genotypes. *N* = 1600. (b) lnRR for four *A. thaliana* genotypes in two‐way mixtures with each of the other three genotypes in the presence and absence of *Hpa*. X axis: focal genotypes are indicated, and all other genotypes are neighbours. *N* = 1600. Error bars show 95% confidence interval of means.

Competitive intensity, estimated by lnRR of genotypes in two‐way mixtures was altered by the presence of the pathogen, as shown by a significant interaction between genotype, cultivation method (two‐way mixture or monoculture) and the presence or absence of *Hpa* (Fig. 1a; Table S2, *F*_4,23_ = 3·54, *P *=* *0·007). The outcome of specific competitive interactions in the two‐way mixtures was heavily dependent on the pathogen (Fig. 1b). *Hpa* reduced seed production in the most susceptible genotypes, NFA‐8 and NFA‐10 (Fig. S1a). This was associated with reductions in rosette diameter (Fig. S1b) and lnRR (Fig. 1b). By reducing fitness of susceptible genotypes, *Hpa* indirectly increased the competitive ability of the more resistant genotypes, Ga‐0 and Van‐0, in two‐way and four‐way mixtures where they had higher lnRR, when they were attacked by the pathogen (Fig. 1a).

**Figure 2:**
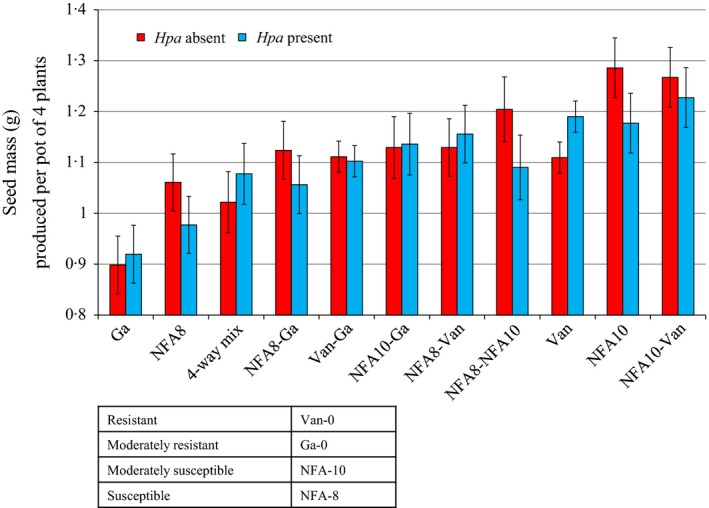
Mean seed production (g) per pot of four *Arabidopsis thaliana* plants comprising one, two or four genotypes and in the presence and absence of *Hyaloperonospora arabidopsidis*. *N* = 1600. Error bars show 95% confidence interval of means.

**Figure 3:**
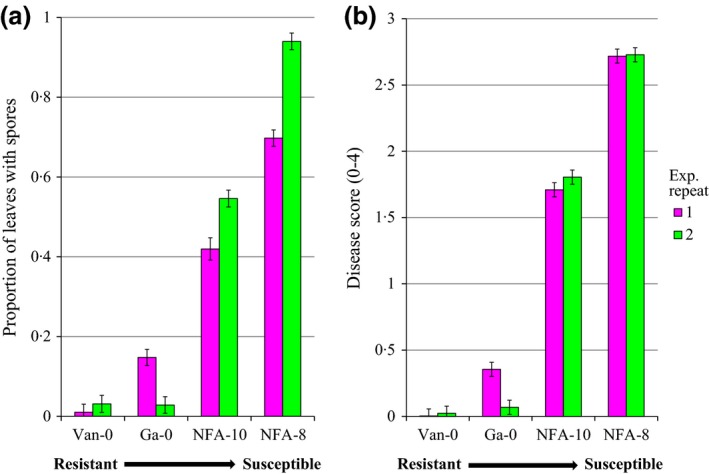
Mean disease scores for individual *Arabidopsis thaliana* plants infected with *Hyaloperonospora arabidopsidis* in experimental repeats 1 and 2. (a) Proportion of leaves showing signs of sporulation 6 days post‐inoculation. (b) Disease score (0 = no disease, 4 = over 67% leaf area covered in spores) 10 days post‐infection. *N* = 1600. Error bars show 95% confidence interval of means.

Certain combinations of two genotypes produced significantly more seed than monocultures of either component genotype in the presence or absence of *Hpa* or both (Fig. 2; Table S2, *F*_4,23_ = 3·54, *P *=* *0·007). Genotypes that consistently over‐yielded in mixture were identified by positive values of lnRR (Fig. 1), and classed as highly competitive. The partially resistant Ga‐0 was the most competitive genotype whether *Hpa* was present or not (Fig. 1a). NFA‐8 was highly competitive in the absence of the pathogen, but not in its presence (Fig. 1) owing to its high susceptibility to *Hpa* (Fig. 3). Treatments containing only these highly competitive genotypes were the lowest yielding overall, whereas pots containing less competitive genotypes, the moderately susceptible NFA‐10 and the fully resistant Van‐0, were the highest yielding overall, in both monoculture and the respective two‐way mixture (Fig. 2). Compensatory dynamics (indicated by negative covariances of seed mass in mixture) for certain pairs of genotypes was observed more often than not in the presence of *Hpa* (Table 1). Some genotype combinations, such as NFA‐8 with NFA‐10 and Van‐0 with Ga‐0, showed strong correlated dynamics, indicated by positive covariances, in both the presence and absence of *Hpa* indicating complementation, facilitation, both processes or simply a lack of competition (Table 1).

**Table 1:** Mean covariances in seed production between two *Arabidopsis thaliana* genotypes grown together in the same pot (2 plants/genotype/pot = a total of 4 plants/pot) in the presence and absence of *Hyaloperonospora arabidopsidis* (*Hpa*)

*Hpa*	Genotype	Ga‐0	NFA‐10	NFA‐8	Van‐0
Absent	Ga‐0	–	−3·69E‐04	8·35E‐05	1·59E‐02
Absent	NFA‐10	–	–	7·05E‐03	6·96E‐03
Absent	NFA‐8	–	–	–	7·99E‐04
Absent	Van‐0	–	–	–	–
Present	Ga‐0	–	−3·85E‐03	−1·01E‐02	3·88E‐03
Present	NFA‐10	–	–	7·34E‐04	−6·29E‐03
Present	NFA‐8	–	–	–	−1·39E‐02
Present	Van‐0	–	–	–	–

Negative values indicate compensatory dynamics and positive values indicate correlated dynamics. *N* = 1600.

Disease (Fig. 3), rosette size, flowering time, seed production and consequently competitive ability (lnRR) varied between experiments (Fig. S2). Variation in environmental conditions in the glasshouse affected disease progress (Fig. 3) and plant development (Fig. S2a,b). The proportion of leaves with sporulation 6 dpi was higher for NFA‐8 and NFA‐10 in the second experiment than in the first, although they remained the most susceptible genotypes (Fig. 3a; Table S3a, *F*_3,8_ = 35·43, *P *<* *0·001). By contrast, there was no overall significant difference between experimental repeats for disease scores at 10 dpi indicating that by this stage the pathogen had achieved maximum disease levels (Fig. 3b; Table S3b, *F*_1,8_ = 0·85, *P* = 0·4) although Ga‐0 was more resistant in the second experiment at both 6 and 10 dpi (Fig. 3). In the second experiment, rosette diameter was greater after 5 weeks growth (Fig. S2a; Table S4, *F*_1,16_ = 2145·41, *P *<* *0·001), and the number of days to flower fewer (Fig. S2b; Table S5, *F*_1,30_ = 3265·15, *P *<* *0·001), which ultimately led to increased seed production (Fig. S2c; Table S2, *F*_1,23_ = 209·68, *P* < 0·001). Competitive ability (lnRR) and the outcomes of competition under each treatment were fairly consistent between replicates despite minor variation (Fig. S2d; Table S2, *F*_4,23_ = 3·54, *P* = 0·007).

On average across treatments and genotypes, two‐way mixtures achieved greater yields than monocultures and four‐way mixtures (Fig. S3a; Table S6, *F*_2,31_ = 6·76, *P* = 0·001). Four‐way mixtures produced the lowest yields in the absence of the pathogen, and yields similar to the mean of monoculture yields in the presence of *Hpa*. Yield variability was estimated by the SD of log(seed mass) after adjusting for the effects of experiment and disease treatment by multiple linear regression. The lowest yield variability, that is the greatest yield stability, was obtained in the two‐way mixtures, which had 13% lower SD than the four‐way mixtures (*P* < 0·001) and 16% lower SD than monocultures (*P* < 0·001). A likely cause was high inter‐plant competition resulting from the presence of NFA‐8 and particularly Ga‐0. Seed production decreased as genotypic diversity increased for the less competitive genotypes Van‐0 (fully resistant) in the absence of *Hpa*, and NFA‐10 (moderately susceptible) in the presence of *Hpa* (Fig. S3b; Table S6, *F*_8,31_ = 2·44, *P *=* *0·01). Ga‐0 was the only genotype to overyield significantly in the four‐way mixture compared to monoculture in the presence of *Hpa*, further illustrating its stronger competitive ability (Fig. S3b, *P < *0·01). Both genotypic diversity and composition contributed to competitive intensity between plants, ultimately affecting yield and yield stability.

### Turnip Yellows Virus

**Figure 4:**
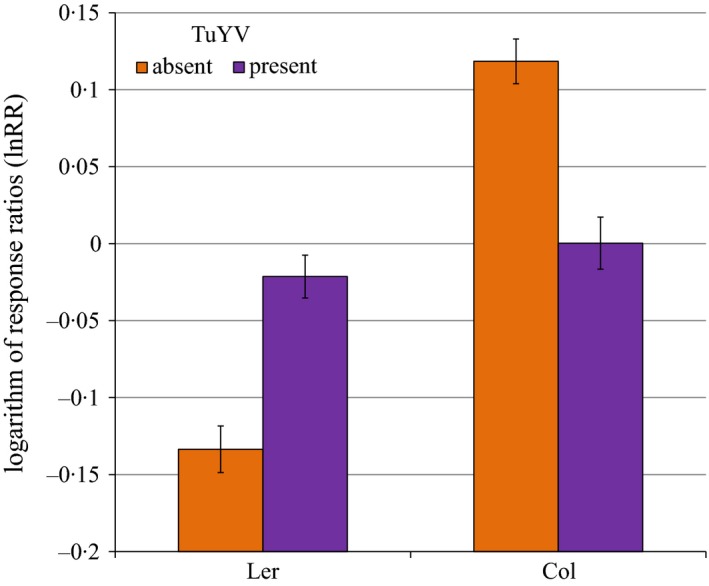
Competitive intensity measured by the logarithm of response ratios (lnRR) (log seed in mixture/seed in monoculture) of two *Arabidopsis thaliana* genotypes in the presence and absence of *Turnip yellows virus* (TuYV). *N* = 400. Error bars show 95% confidence interval of means.

Col‐0 and Ler‐1 were susceptible to TuYV, allowing virus titre to reach similar levels in both genotypes (Fig. S4) and observed visually by purpling of leaves (Fig. S5). Both genotypes had delayed flowering time in mixtures compared to monocultures in the presence of TuYV, but the delay was greatest for infected Col‐0 (Fig. S6a; Table S7, *F*_2,7_ = 4·12, *P *=* *0·02). Ler‐1 produced a larger rosette after 5 weeks growth in monoculture than in the mixture, possibly due to higher inter‐plant competition in the mixture (Fig. S6b; Table S8, *F*_1,4_ = 3·96, *P *=* *0·05). The more competitive genotype, Col‐0, overyielded in uninfected mixtures at the expense of Ler‐1, which produced less seed (Fig. S6c; Table S9, *F*_2,7_ = 6·58, *P *=* *0·002). However, when the virus was present both genotypes performed as well in mixture as they did in monoculture, due to a large reduction in the competitive ability of Col‐0 (Fig. 4). Despite changes in competitive ability due to pathogen infection, the average yield in mixtures and the average of the monocultures was stable whether the pathogen was present or absent, and there was no overall yield penalty as a result of growing mixtures (Fig. S7).

## Discussion

This study demonstrates the importance of pathogen‐mediated competition in maintaining plant genotypic diversity and productivity. The use of *A. thaliana* as a model system has enabled tests of hypotheses about the mechanism by which disease maintains genotypic diversity in plant populations and the role of genotypic diversity in maintaining or enhancing plant productivity at the population level. The experiments reported here identified compensatory competitive interactions, in which overyielding by some genotypes compensated for the loss of yield by others, as the main mechanisms leading to increased stability and productivity of genotypically and phenotypically diverse plant populations under disease pressure. Compensatory interactions were greatest when genotypes with different competitive abilities were combined in the presence of the pathogen. This investigation supports both the diversity–productivity hypothesis (Darwin 1872) and the diversity–stability hypothesis (Elton 1958) which state that greater plant diversity can lead to an increase in productivity or stability, respectively. A key finding from this study is that the composition of the plant population, specifically the presence and maintenance of resistant genotypes within that population, is responsible for the capacity of the population to maintain productivity, stability and diversity.

Susceptible genotypes suffered greatly in the presence of either pathogen owing to reduced plant fitness and competitive ability. This led to reduced seed production in susceptible monocultures but in mixtures containing genotypic variation for disease susceptibility, a reduction in competitive ability of susceptible genotypes allowed increased seed production by more resistant or tolerant genotypes. Increased productivity with greater diversity may be the result of an increased likelihood of a productive species being present (sampling effect) and from a greater chance of efficient exploitation of all available niches (complementation)(Tilman 1996; Hector *et al*. 1999). The results reported here provide experimental support for the hypothesis that competitive interactions also play a significant role in driving the diversity–productivity relationship (Schmid 1994; Tilman, Wedin & Knops 1996; Hector *et al*. 1999). Evidence for plant diversity enhancing yield and yield stability under disease pressure is supported by this study and by several studies of natural systems in which plant pathogens promoted biodiversity by preventing competitive exclusion and encouraging complementation (Bradley, Gilbert & Martiny 2008; Maron *et al*. 2011). Cultivating plants as mixtures may reduce pathogen‐constrained root growth compared to monocultures and lead to apparent competitive release in above‐ground parts because genotype‐specific soil pathogens that accumulate in monocultures are diluted (de Kroon *et al*. 2012). *Arabidopsis thaliana* genotypes can grow larger in sterile soil (soil pathogens absent) compared to non‐sterile soil but the amount of ‘enemy release’ appears independent of the competing plant genotype (Aguilera *et al*. 2011).

Genotypic diversity enhanced ecological resistance of the plant population to attack by *H. arabidopsidis* as shown by an increase in yield and yield stability in two‐way mixtures compared to the average of the component monocultures or four‐way mixtures; ecological resistance refers to the ability of the system to resist change following perturbation (Pimm 1984) and is not the same as resistance of individual plants to disease. Four‐way mixtures produced the lowest yields in the absence of *Hpa*, apparently because of the presence of two highly competitive genotypes (Ga‐0 and NFA‐8) that outcompeted neighbours for resources and overyielded in mixture, yet produced less seed than more weakly competing genotypes. Thus, mixture composition rather than genotypic diversity *per se* affected productivity in this study. This contrasts with several studies on cereals showing a trend towards greater yields with more varieties in the mixture, largely because of superior disease control (Newton *et al*. 1997; Newton, Hackett & Swanston 2008). However, modern cereal varieties are more genetically and phenotypically similar than *A. thaliana* accessions and therefore vary less in competitive ability. The highest yielding two‐way mixtures consisted of genotypes with relatively low competitive abilities and greater investment in reproductive effort (Van‐0 and NFA‐10), providing evidence for a fundamental tenet of life‐history theory that reproduction is costly and results in trade‐offs with other fitness components, regularly observed in crop plants (Lemerle *et al*. 2006; Song *et al*. 2010).

Compensatory interactions, where decreased productivity of some genotypes led to increased productivity of others, occurred in both pathosystems and buffered against pathogen‐induced alterations to the competitive ability of the host population. This resulted in yield stability of the mixed genotype population, supporting results from both natural systems (Tilman 1996) and agriculture (Finckh *et al*. 2000). By contrast, the pathosystems differed in the effect of host genotype mixtures on productivity. Mixtures had a yield advantage when plants were inoculated with *Hpa,* but not with TuYV. This contrast may be a feature of the disease, the plant genotypes used, or both. In the absence of TuYV, the higher yielding Col‐0 genotype maintained productivity in monoculture despite higher inter‐plant competition than in a mixture with Ler‐1. This contrasts with the *Hpa* experiment in which the highly competitive genotypes produced significantly less seed in non‐diseased monocultures. A possible explanation for this difference is that the most competitive genotype in the TuYV experiment was less competitive than the most competitive genotypes in the *Hpa* experiment and therefore was under less competition in monoculture. A significant conclusion from a comparison of experiments on the two pathogens is that the outcome of competition depends on the effect of the pathogen on host fitness and the identity of the plant genotypes present in the mixture, not simply the number of host genotypes.

Within‐plant compensation was observed in the TuYV experiments as Ler‐1 was able to maintain seed production in mixture despite reduced rosette size through alteration of resource allocation. No signs of within‐plant compensation were observed in the *Hpa* experiment possibly due to the pathogen isolate or plant genotypes used. This highlights the fact that different pathogens interact with hosts in different ways (Jones & Dangl 2006) and that successful plant genotypic mixtures must confer resistance to multiple pathogens. The delayed flowering time of both genotypes in mixtures compared to monocultures indicates an alteration of plant development strategy in response to stress caused by the combination of plant competition and the pathogen. Plants may delay or accelerate the reproductive transition in response to disease (Korves & Bergelson 2003) while life‐history evolution studies predict that organisms at risk of severe disease will evolve fast reproduction strategies to reduce damage from parasites (Forbes 1993; Agnew, Koella & Michalakis 2000). Delaying the reproductive transition can allow greater investment in vegetative growth, thus increasing seed production (Bazzaz *et al*. 1987) but may also have the opposite effect (Kudoh *et al*. 2002). Here, the direct effect of altered flowering time on yield could not be determined as the effects of the pathogen on seed production and flowering time could not be separated. This study indicates that host plant responses to pathogens can vary greatly depending on their interaction, which must be considered when assessing and predicting plant population responses to multiple pathogens. Genotypic mixtures led to yield stability in both infected and uninfected populations through a combination of altered plant resource allocation and overyielding by fitter genotypes.

Understanding the mechanisms of plant competition increases the predictability of the outcome of competition for different resources. Knowledge of plant–plant interactions contributing to such mechanisms can facilitate exploitation of plant genotypic diversity, stabilizing productivity by increasing the efficiency of the deployment of genotype mixtures in agriculture (Knott & Mundt 1990). Mixtures containing diversity for important functional traits relating to competitive ability (Cahill, Kembel & Gustafson 2005; Creissen, Jorgensen & Brown 2013) and response to environmental stresses such as drought (van Ruijven & Berendse 2010), herbivory (Kotowska, Cahill & Keddie 2010) and disease (Mundt 2002) are predicted to have greater ecological resistance and achieve greater yield and yield stability in variable environments through ecological processes including compensatory interactions, complementation and facilitation. This study has indicated ecological mechanisms by which diverse plant populations buffer against disease, achieving high, stable yields and ecological resistance to pathogen attack.

This study provided an experimental test of theories in plant ecology about the effect of pathogens on plant fitness, competitive ability and population diversity. It did not fully support the widely held hypothesis that diversity increases productivity because plant genotypic composition rather than genotypic diversity had the greatest effect on yield and yield stability. Whereas pathogens reduced the fitness of some genotypes, the decrease in competition allowed others to increase productivity and these compensatory interactions led to yield stability in the mixtures. By increasing knowledge of natural processes operating in diverse plant populations, this study demonstrates methods that can inform decisions about suitable plant cultivars for cultivation as mixtures.

## Supporting information


**Lay Summary**

**Fig. S1** Fitness of four *Arabidopsis thaliana* genotypes grown in monoculture and 2‐way genotype mixtures and in the presence and absence of *Hyaloperonospora arabidopsidis* (*Hpa*).

**Fig. S2** Phenotypic fitness measurements taken for four *Arabidopsis thaliana* genotypes grown in experimental repeats 1 and 2 and in the presence and absence of *Hyaloperonospora arabidopsidis* (*Hpa*).

**Fig. S3** Mean seed mass yields for *Arabidopsis thaliana* plants grown as 1, 2 or 4 genotypes per pot in the presence or absence of *Hyaloperonospora arabidopsidis* (*Hpa*).

**Fig. S4** Enzyme‐linked immunosorbent assay detection of *Turnip yellows virus* (TuYV) for two *Arabidopsis thaliana* genotypes.

**Fig. S5** Photographs of *Turnip yellows virus* (TuYV) infected *Arabidopsis thaliana* after 10 weeks growth.

**Fig. S6** Phenotypic fitness measurements taken for two *Arabidopsis thaliana* genotypes grown in the presence and absence of *Turnip yellows virus* (TuYV).

**Fig. S7** Mean seed production (g) per pot of four *Arabidopsis thaliana* genotypes grown in monoculture and mixture and in the presence and absence of *Turnip yellows virus* (TuYV). *N* = 400.

**Table S1** (a) Mean trait values for four *Arabidopsis thaliana* genotypes grown in the absence of competition and the presence or absence of *Hyaloperonospora arabidopsidis* (*Hpa*). (b) Mean trait values for two *Arabidopsis thaliana* genotypes grown in the absence of competition and the presence or absence of *Turnip yellows virus* (TuYV).

**Table S2** Results from linear mixed modelling to evaluate the effect of *Arabidopsis thaliana* genotypic diversity and *Hyaloperonospora arabidopsidis* (*Hpa*) on seed mass produced per plant in a pair‐wise interaction experiment.

**Table S3** (a) Results from linear mixed modelling to evaluate the effect of *Arabidopsis thaliana* genotypic diversity and *Hyaloperonospora arabidopsidis* (*Hpa*) on the initial disease score at 6 days after infection in a pair‐wise interaction experiment. (b) Results from linear mixed modelling to evaluate the effect of *Arabidopsis thaliana* genotypic diversity and *Hyaloperonospora arabidopsidis* (*Hpa*) on the second disease score at 10 days after infection in a pair‐wise interaction experiment.

**Table S4** Results from linear mixed modelling to evaluate the effect of *Arabidopsis thaliana* genotypic diversity and *Hyaloperonospora arabidopsidis* (*Hpa*) on rosette diameter in a pair‐wise interaction.

**Table S5** Results from linear mixed modelling to evaluate the effect of *Arabidopsis thaliana* genotypic diversity and *Hyaloperonospora arabidopsidis* (*Hpa*) on days to flower in a pair‐wise interaction experiment.

**Table S6** Results from linear mixed modelling to evaluate the effect of *Arabidopsis thaliana* genotypic diversity and *Hyaloperonospora arabidopsidis* (*Hpa*) on seed productivity in a competitive interaction experiment.

**Table S7** Results from linear mixed modelling to evaluate the effect of *Arabidopsis thaliana* genotypic diversity and *Turnip yellows virus* (TuYV) on days to flower in a pair‐wise interaction experiment.

**Table S8** Results from linear mixed modelling to evaluate the effect of *Arabidopsis thaliana* genotypic diversity and *Turnip yellows virus* (TuYV) on rosette diameter in a pair‐wise interaction experiment.

**Table S9** Results from linear mixed modelling to evaluate the effect of *Arabidopsis thaliana* genotypic diversity and *Turnip yellows virus* (TuYV) on seed productivity in a pair‐wise interaction experiment.
