# Supplementary material for: Impact of disease on diversity and productivity of plant populations
Source: Funct Ecol. 2015 Sep 23;30(4):649–57. doi: 10.1111/1365-2435.12552 (PMC4974914; doi:10.1111/1365-2435.12552)
Supplement: Supplementary file 1 — Lay Summary [file FEC-30-649-s001.pdf]

Functional Ecology
British Ecological Society

Every plant needs good neighbours

Henry E. Creissen, Tove H. Jorgensen and James K.M. Brown

The relationship between plant diversity, ecological stability and ecosystem productivity has been studied extensively in recent decades. Plant herbivores, diseases and insect pests alter such relationships by affecting plant fitness, reducing the growth and competitive ability of diseased plants. This can impact heavily upon plant population and community structure. Yet, despite their importance, experimental tests of mechanisms of pathogen-induced changes to plant competitive ability, productivity and diversity are rare.

We conducted competition experiments involving different genotypes of a small plant *(Arabidopsis thaliana)* to examine the relationships between population diversity, composition, productivity and stability in diseased and non-diseased plant populations. Plant genotypes varied greatly in both competitive ability and response to two pathogens, a virus and an oomycete. In certain populations we observed compensatory competitive interactions in which some genotypes increased productivity and compensated for the loss of yield by others. These interactions ultimately resulted in increased stability and productivity of the population as a whole. This study shows the importance of pathogen-mediated competition in maintaining plant genotypic diversity and productivity. A key finding is that the genotypic composition of the plant population, specifically the presence and maintenance of resistant genotypes within that population, is responsible for the capacity of the population to maintain productivity, stability and diversity.

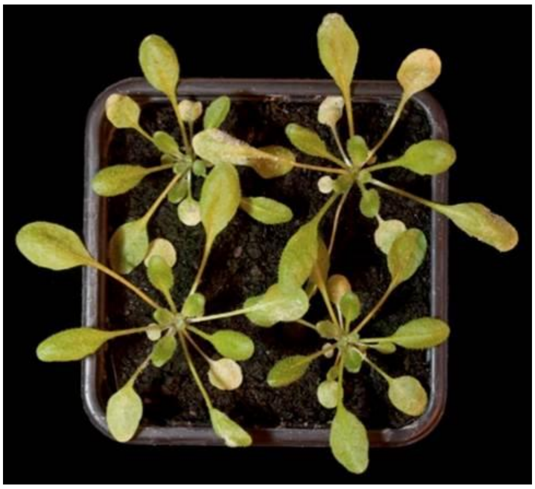

Four different Arabidopsis genotypes competing whilst under disease pressure.

As well as increasing our understanding of natural processes occurring in genotypically and phenotypically diverse plant populations, this study has potential applications to agriculture by demonstrating methods that can inform decisions about suitable plant cultivars for cultivation as variety mixtures.
