# Supplementary material for: Impact of disease on diversity and productivity of plant populations
Source: Funct Ecol. 2015 Sep 23;30(4):649–57. doi: 10.1111/1365-2435.12552 (PMC4974914; doi:10.1111/1365-2435.12552)

Fig. S1. Fitness of four *Arabidopsis thaliana* genotypes grown in monoculture and 2-way genotype mixtures and in the presence and absence of *Hyaloperonospora arabidopsidis* (*Hpa*). a) Reproductive fitness indicated by mean seed mass produced per plant. b) Vegetative fitness indicated by mean rosette diameter. Error bars show 95% confidence interval of mean. N=1600.

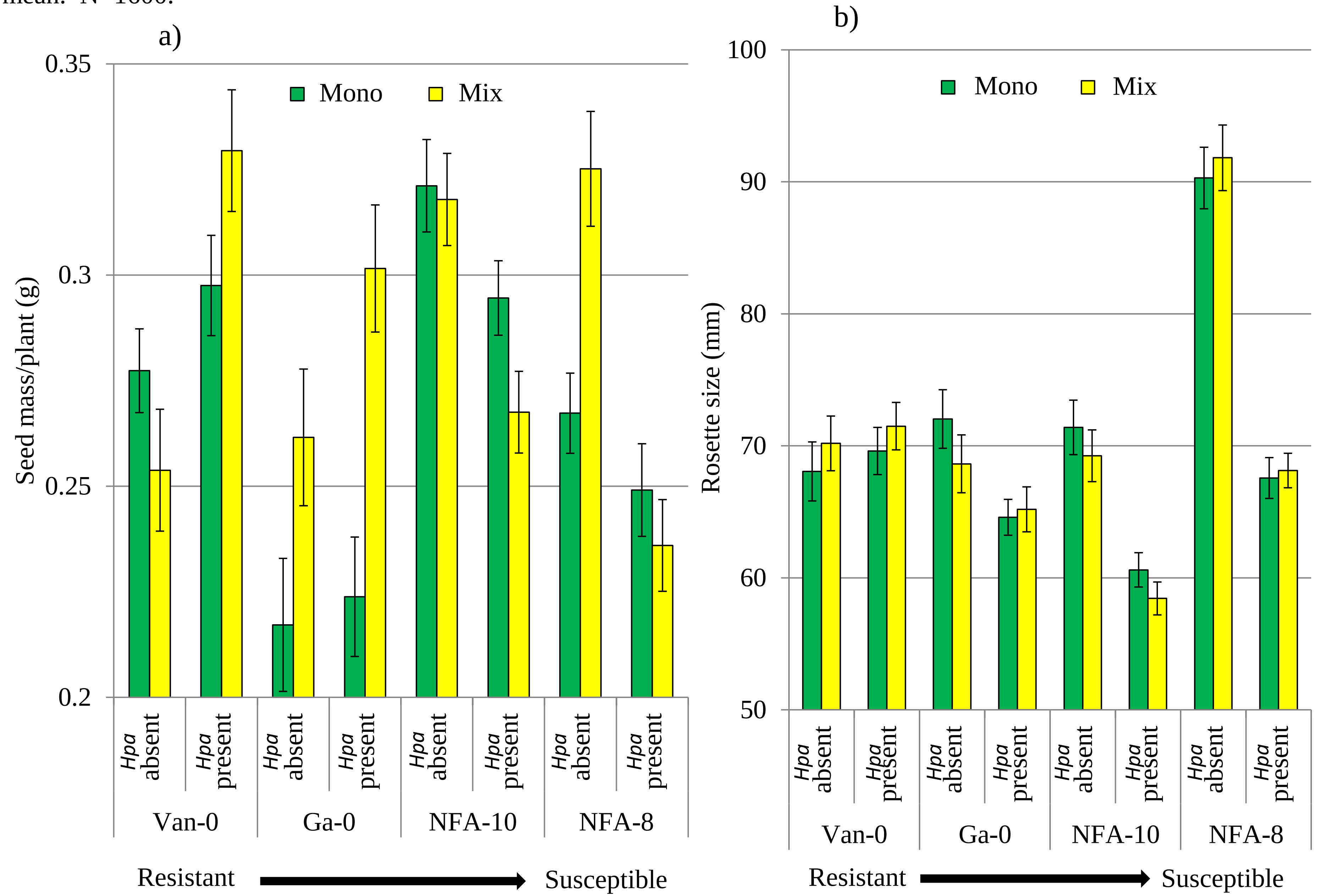

Supplement: Supplementary file 2 — Fig. S1 Fitness of four Arabidopsis thaliana genotypes grown in monoculture and 2‐way genotype mixtures and in the presence and absence of Hyaloperonospora arabidopsidis (Hpa). [file FEC-30-649-s002.pdf]
