# Supplementary material for: Impact of disease on diversity and productivity of plant populations
Source: Funct Ecol. 2015 Sep 23;30(4):649–57. doi: 10.1111/1365-2435.12552 (PMC4974914; doi:10.1111/1365-2435.12552)

Fig. S2. Phenotypic fitness measurements taken for four *Arabidopsis thaliana* genotypes grown in experimental repeats 1 and 2 and in the presence and absence of *Hyaloperonospora arabidopsidis* (*Hpa*). **a)** Mean rosette diameter after five weeks growth. **b)** Mean number of days taken to flower. **c)** Mean seed mass produced per plant averaged over all four genotypes. **d)** The logarithm of response ratios (lnRR) an indicator of competitive ability. Error bars show 95% confidence interval of mean.

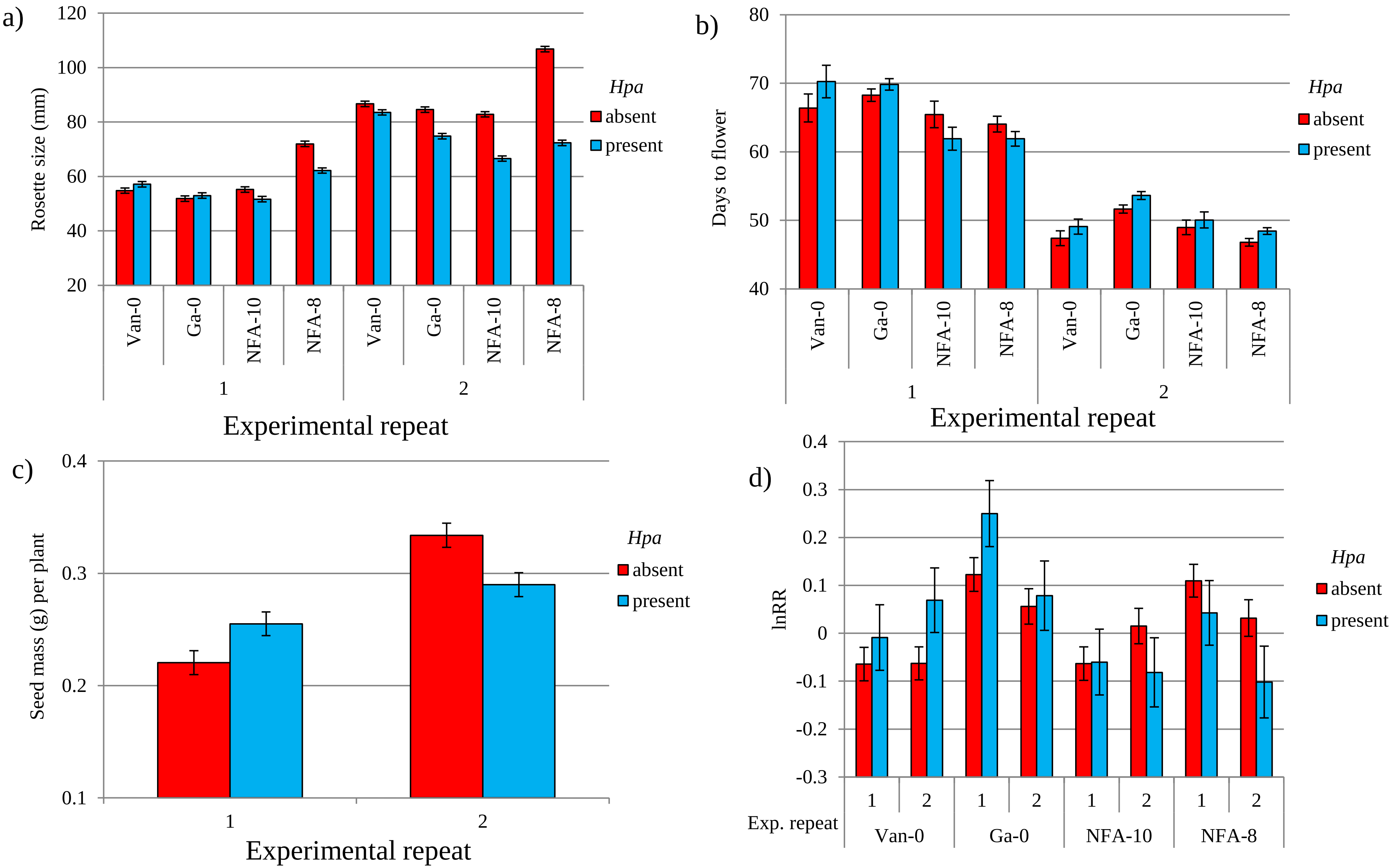

Supplement: Supplementary file 3 — Fig. S2 Phenotypic fitness measurements taken for four Arabidopsis thaliana genotypes grown in experimental repeats 1 and 2 and in the presence and absence of Hyaloperonospora arabidopsidis (Hpa). [file FEC-30-649-s003.pdf]
