# Supplementary material for: Impact of disease on diversity and productivity of plant populations
Source: Funct Ecol. 2015 Sep 23;30(4):649–57. doi: 10.1111/1365-2435.12552 (PMC4974914; doi:10.1111/1365-2435.12552)

**Fig. S3. a)** Mean seed mass yields for *Arabidopsis thaliana* plants grown as 1, 2 or 4 genotypes per pot in the presence or absence of *Hyaloperonospora arabidopsidis* (*Hpa*). N=1600. Error bars show 95% confidence interval of means. **b)** Mean seed mass produced per plant for each genotype in pots containing 1, 2 or 4 genotypes in the presence and absence of *Hpa*. N=1600. Error bars show 95% confidence interval of means.

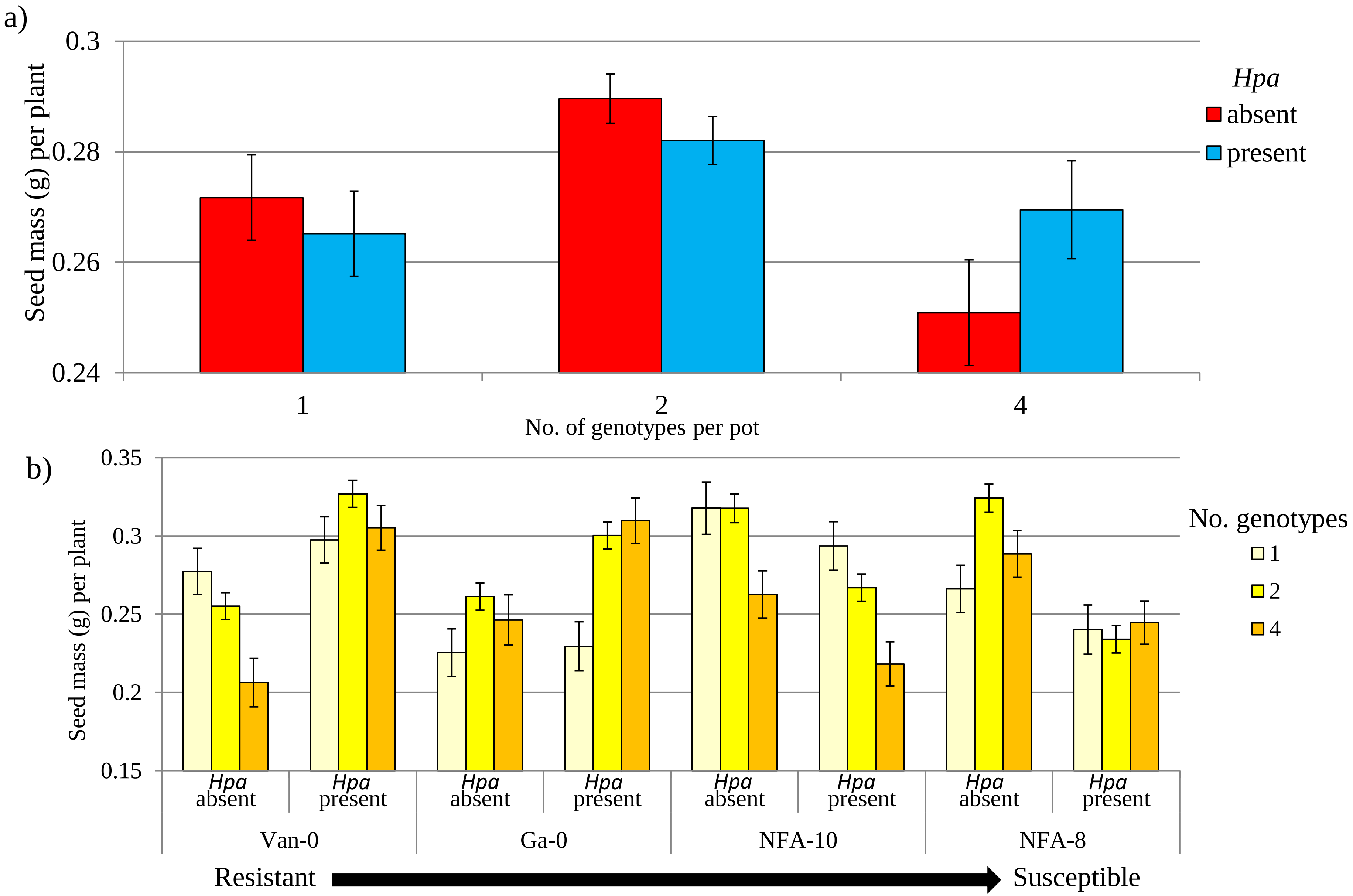

Supplement: Supplementary file 4 — Fig. S3 Mean seed mass yields for Arabidopsis thaliana plants grown as 1, 2 or 4 genotypes per pot in the presence or absence of Hyaloperonospora arabidopsidis (Hpa). [file FEC-30-649-s004.pdf]
