# Supplementary material for: Impact of disease on diversity and productivity of plant populations
Source: Funct Ecol. 2015 Sep 23;30(4):649–57. doi: 10.1111/1365-2435.12552 (PMC4974914; doi:10.1111/1365-2435.12552)

**Fig. S4.** Enzyme-linked immunosorbent assay (ELISA) detection of *Turnip yellows virus* for two *Arabidopsis thaliana* genotypes measured as absorbance at 405 nm five weeks post inoculation.  N=400. Error bars show 95% confidence interval of means.

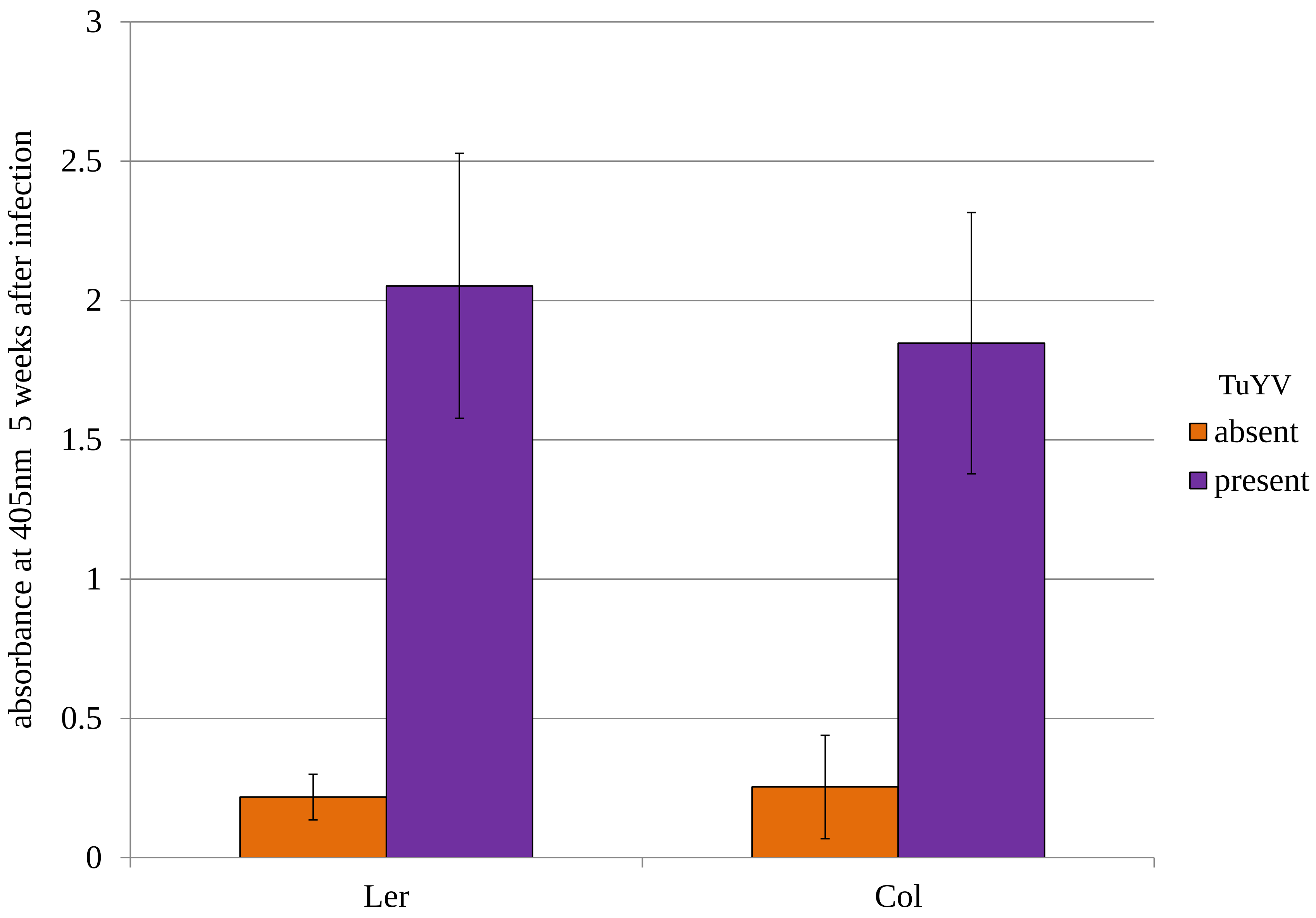

Supplement: Supplementary file 5 — Fig. S4 Enzyme‐linked immunosorbent assay detection of Turnip yellows virus (TuYV) for two Arabidopsis thaliana genotypes. [file FEC-30-649-s005.pdf]
