# Supplementary material for: Impact of disease on diversity and productivity of plant populations
Source: Funct Ecol. 2015 Sep 23;30(4):649–57. doi: 10.1111/1365-2435.12552 (PMC4974914; doi:10.1111/1365-2435.12552)

Fig. S5. Photographs of *Turnip yellows virus* (TuYV) infected *Arabidopsis thaliana* after ten weeks growth. **a)** Monoculture of four Col-0 plants. **b)** Monoculture of four Ler-1 plants.

a)

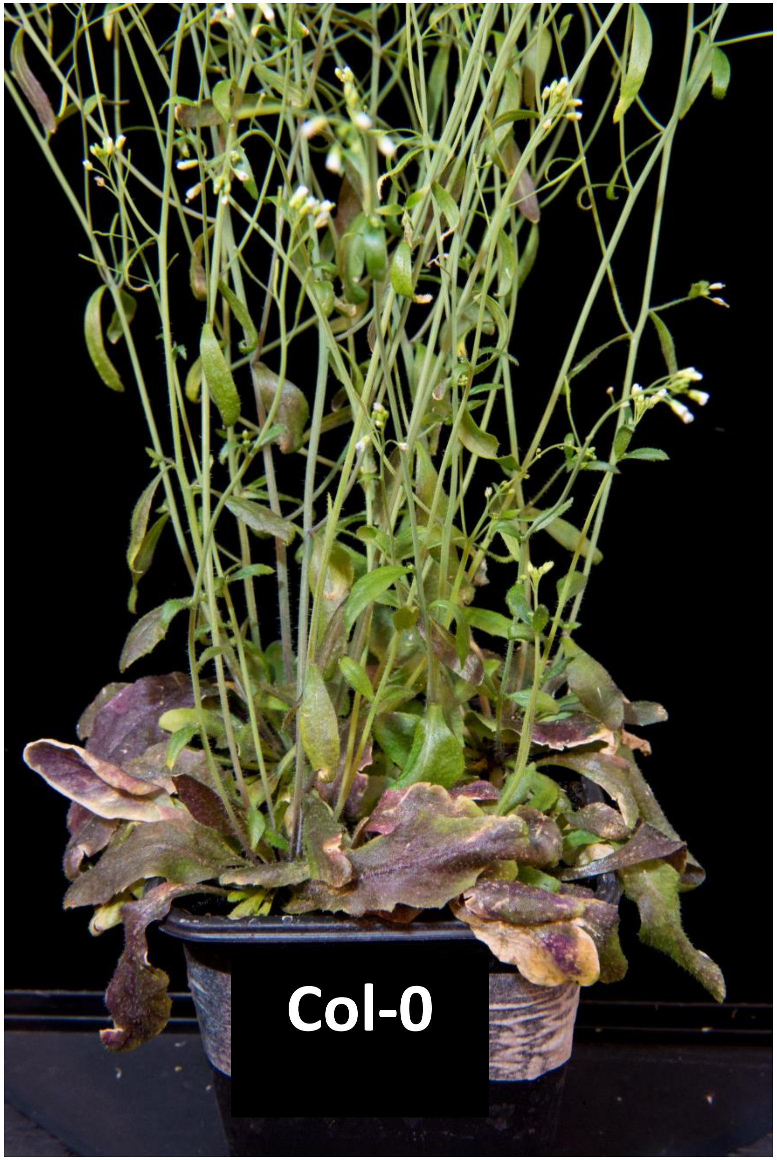

b)

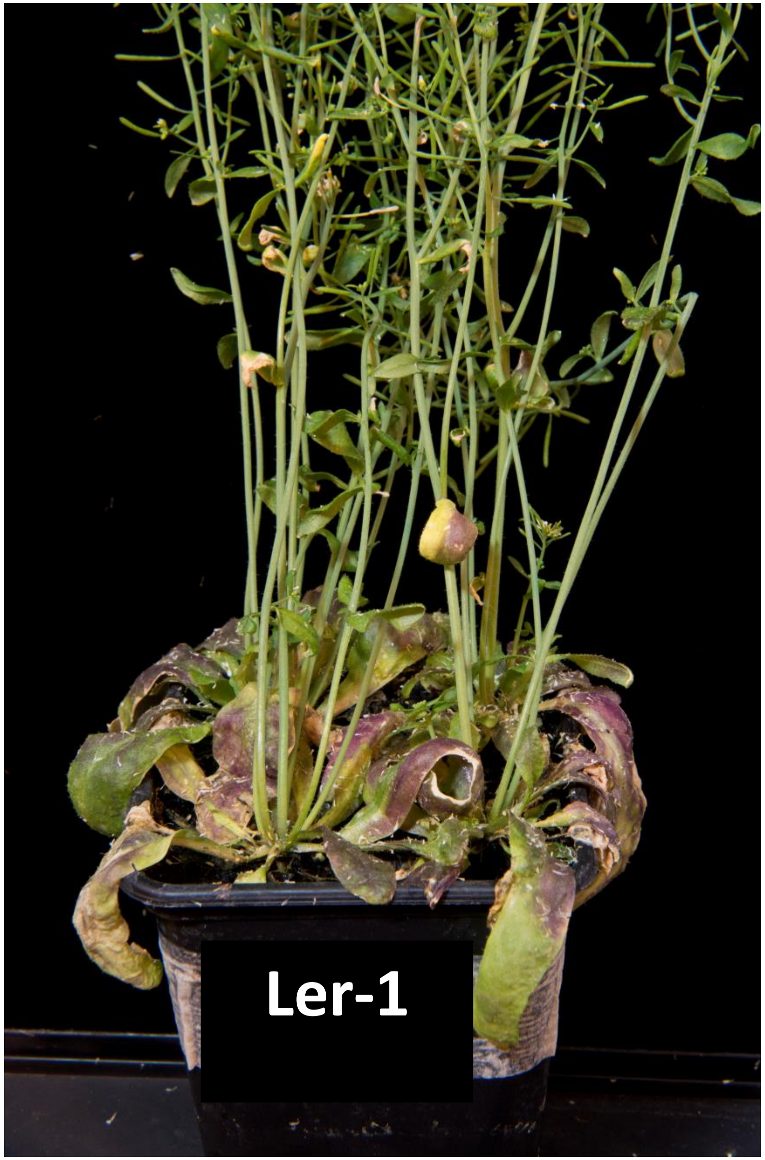

Supplement: Supplementary file 6 — Fig. S5 Photographs of Turnip yellows virus (TuYV) infected Arabidopsis thaliana after 10 weeks growth. [file FEC-30-649-s006.pdf]
