# Supplementary material for: Impact of disease on diversity and productivity of plant populations
Source: Funct Ecol. 2015 Sep 23;30(4):649–57. doi: 10.1111/1365-2435.12552 (PMC4974914; doi:10.1111/1365-2435.12552)

Fig. S6. Phenotypic fitness measurements taken for two *Arabidopsis thaliana* genotypes grown in the presence and absence of *Turnip yellows virus* (TuYV). **a)** Mean number of days taken to flower. **b)** Mean rosette diameter after five weeks growth. **c)** Mean seed mass produced. N=400. Error bars show 95% confidence interval of means.

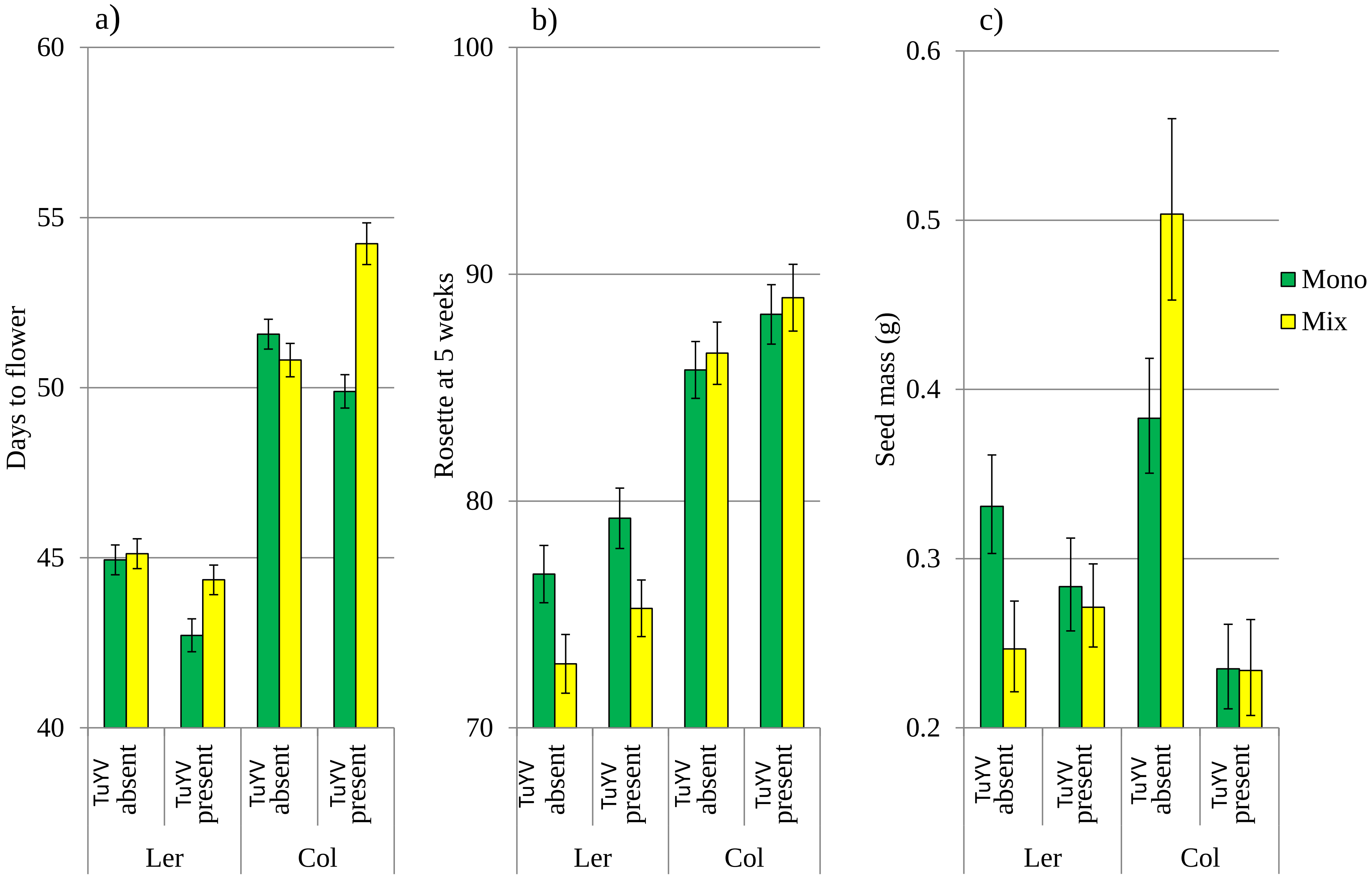

Supplement: Supplementary file 7 — Fig. S6 Phenotypic fitness measurements taken for two Arabidopsis thaliana genotypes grown in the presence and absence of Turnip yellows virus (TuYV). [file FEC-30-649-s007.pdf]
