# Supplementary material for: Impact of disease on diversity and productivity of plant populations
Source: Funct Ecol. 2015 Sep 23;30(4):649–57. doi: 10.1111/1365-2435.12552 (PMC4974914; doi:10.1111/1365-2435.12552)

Fig. S7. Mean seed production (g) per pot of four *Arabidopsis thaliana* genotypes grown in monoculture and mixture and in the presence and absence of *Turnip yellows virus* (TuYV). N=400. Error bars show 95% confidence interval of means.

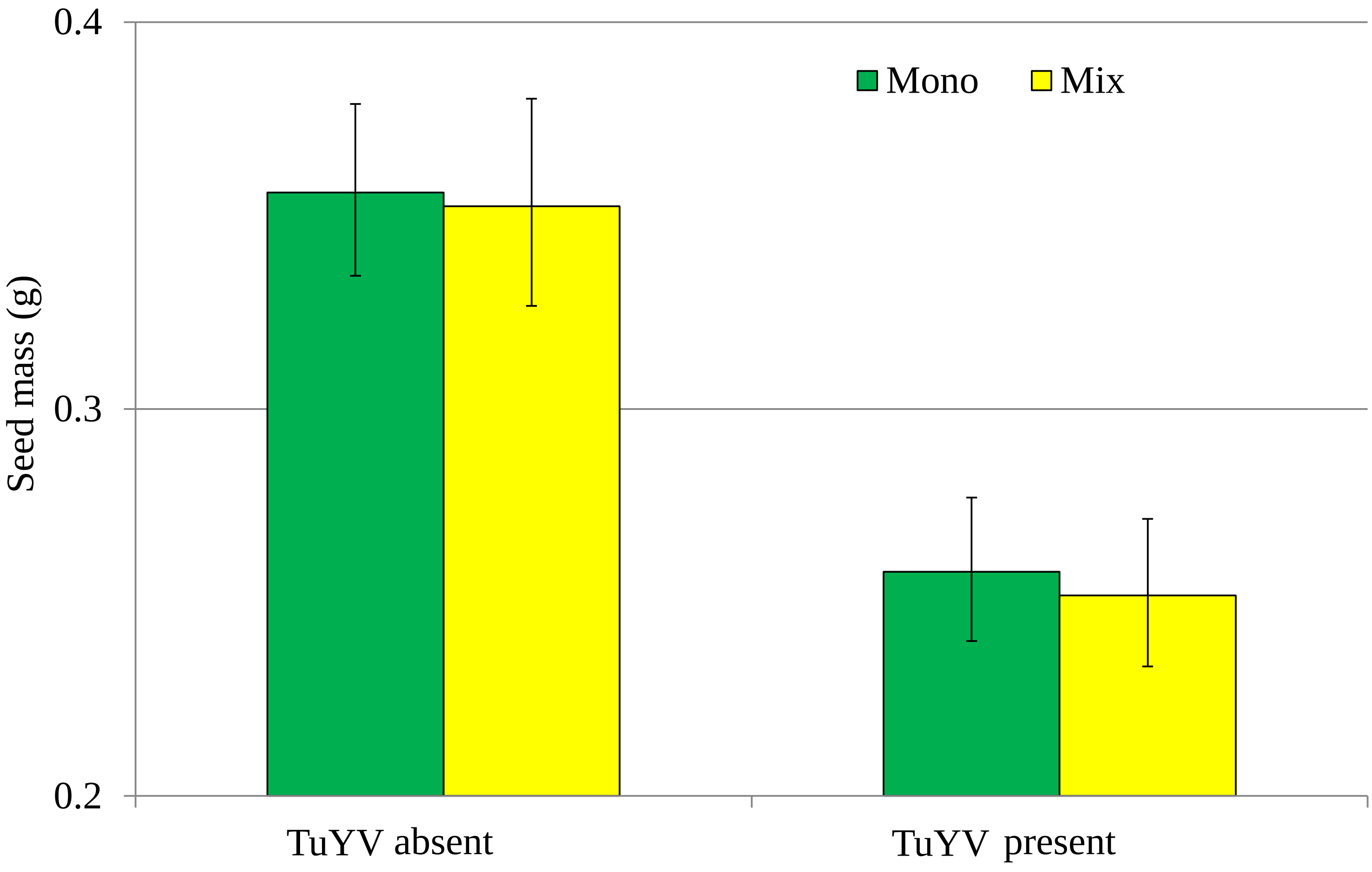

Supplement: Supplementary file 8 — Fig. S7 Mean seed production (g) per pot of four Arabidopsis thaliana genotypes grown in monoculture and mixture and in the presence and absence of Turnip yellows virus (TuYV). N = 400. [file FEC-30-649-s008.pdf]
