# Supplementary material for: Impact of disease on diversity and productivity of plant populations
Source: Funct Ecol. 2015 Sep 23;30(4):649–57. doi: 10.1111/1365-2435.12552 (PMC4974914; doi:10.1111/1365-2435.12552)
Supplement: Supplementary file 9 — Table S1 (a) Mean trait values for four Arabidopsis thaliana genotypes grown in the absence of competition and the presence or absence of Hyaloperonospora arabidopsidis (Hpa). (b) Mean trait values for two Arabidopsis thaliana genotypes grown in the absence of competition and the presence or absence of Turnip yellows virus (TuYV). [file FEC-30-649-s009.pdf]

Table S1a. Mean trait values (± SD) for four *Arabidopsis thaliana* genotypes grown in the absence of competition and the presence or absence of *Hyaloperonospora arabidopsidis* (*Hpa*). N=40 plants/genotype

**Genotype**	**Disease score (0-4)**	**Days taken to flower**		**Rosette diameter at 5 weeks (mm)**		**Seed mass (g)**	
Hpa	present	absent	present	absent	present	absent	present
Van-0	0±0.2	49±9	52±9	72±19	68±14	0.80±0.29	0.78±0.25
Ga-0	0.2±0.5	53±6	57±9	69±17	66±13	0.87±0.31	0.88±0.27
NFA-10	1.8±0.6	57±9	56±7	71±20	61±11	0.93±0.32	1.10±0.33
NFA-8	2.7±0.7	55±9	55±7	88±22	63±7	0.90±0.22	0.78±0.22

Table S1b. Mean trait values (± SD) for two *Arabidopsis thaliana* genotypes grown in the absence of competition and the presence or absence of *Turnip yellows virus* (TuYV). N=40 plants/genotype.

**Genotype**	**Days taken to flower**		**Rosette diameter at 5 weeks (mm)**		**Seed mass (g)**	
TuYV	absent	present	absent	present	absent	present
Col-0	54±2	51±3	91±15	95±15	1.00.±0.28	0.66±0.24
Ler-1	45±3	45±1	89±10	87±	0.72±0.14	0.72.±0.16
