# Supplementary material for: Impact of disease on diversity and productivity of plant populations
Source: Funct Ecol. 2015 Sep 23;30(4):649–57. doi: 10.1111/1365-2435.12552 (PMC4974914; doi:10.1111/1365-2435.12552)
Supplement: Supplementary file 10 — Table S2 Results from linear mixed modelling to evaluate the effect of Arabidopsis thaliana genotypic diversity and Hyaloperonospora arabidopsidis (Hpa) on seed mass produced per plant in a pair‐wise interaction experiment. [file FEC-30-649-s010.pdf]

Table S2. The effect of *Arabidopsis thaliana* genotypic diversity and *Hyaloperonospora arabidopsidis* (*Hpa*) on seed mass produced per plant in a pair-wise interaction experiment. A linear mixed model was used to analyse each factor and all interactions between them. Fixed effects included experimental repeat, genotype, cultivation (2-way mixture/monoculture) and *Hpa* (presence/absence). Non-significant terms were eliminated from the model. *F* and *P* values refer to ANOVA tests of each factor separately and the interactions between them. N=1600.

**Fixed term**	**F**	**n.d.f.**	**d.d.f.**	**P**
Experiment	209.68	1	587.1	<0.001
Genotype	5.69	3	586.7	<0.001
Cultivation method	7.34	1	858.6	0.007
Hpa	0.24	1	586.9	0.621
Experiment. Genotype	86.57	3	587.1	<0.001
Genotype. Cultivation	5.30	3	586.0	<0.001
Experiment. *Hpa*	58.50	1	587.1	<0.001
Genotype. *Hpa*	32.63	3	587.0	<0.001
Genotype. *Hpa*. Experiment	6.00	3	587.2	<0.001
Genotype. *Hpa*. Cultivation	3.54	4	585.5	0.007
