# Supplementary material for: Impact of disease on diversity and productivity of plant populations
Source: Funct Ecol. 2015 Sep 23;30(4):649–57. doi: 10.1111/1365-2435.12552 (PMC4974914; doi:10.1111/1365-2435.12552)
Supplement: Supplementary file 11 — Table S3 (a) Results from linear mixed modelling to evaluate the effect of Arabidopsis thaliana genotypic diversity and Hyaloperonospora arabidopsidis (Hpa) on the initial disease score at 6 days after infection in a pair‐wise interaction experiment. (b) Results from linear mixed modelling to evaluate the effect of Arabidopsis thaliana genotypic diversity and Hyaloperonospora arabidopsidis (Hpa) on the second disease score at 10 days after infection in a pair‐wise interaction experiment. [file FEC-30-649-s011.pdf]

Table S3a. The effect of *Arabidopsis thaliana* genotypic diversity and *Hyaloperonospora arabidopsidis* on the initial disease score at six days after infection in a pair-wise interaction experiment. Disease was scored as the proportion of leaves showing sporulation. A linear mixed model was used to analyse each factor and all interactions between them. Fixed effects included experimental repeat, genotype and cultivation (2-way mixture/monoculture). Non-significant terms were eliminated from the model. *F* and *P* values refer to ANOVA tests of each factor separately and the interactions between them. N=1600.

**Fixed term**	**F**	**n.d.f.**	**d.d.f.**	**P**
Experiment	38.41	1	334.8	<0.001
Genotype	1.79	1	362.5	0.2
Cultivation	664.52	3	634.3	<0.001
Experimental. Genotype	35.43	3	634.0	<0.001

Table S3b. The effect of *Arabidopsis thaliana* genotypic diversity and *Hyaloperonospora arabidopsidis* on the second disease score at ten days after infection in a pair-wise interaction experiment. Disease was scored on a scale of 0-4, with 0=no disease and 4=over 67% of leaf area covered in spores. A linear mixed model was used to analyse each factor and all interactions between them. Fixed effects included experimental repeat, genotype and cultivation (2-way mixture/monoculture). Non-significant terms were eliminated from the model. *F* and *P* values refer to ANOVA tests of each factor separately and the interactions between them. N=1600.

**Fixed term**	**F**	**n.d.f.**	**d.d.f.**	**P**
Experiment	0.85	1	349.2	0.4
Genotype	0.15	1	394.1	0.7
Cultivation	1155.94	3	631.2	<0.001
Experimental. Genotype	4.94	3	631.1	0.002
