# Supplementary material for: Impact of disease on diversity and productivity of plant populations
Source: Funct Ecol. 2015 Sep 23;30(4):649–57. doi: 10.1111/1365-2435.12552 (PMC4974914; doi:10.1111/1365-2435.12552)
Supplement: Supplementary file 12 — Table S4 Results from linear mixed modelling to evaluate the effect of Arabidopsis thaliana genotypic diversity and Hyaloperonospora arabidopsidis (Hpa) on rosette diameter in a pair‐wise interaction. [file FEC-30-649-s012.pdf]

**Table S4.** The effect of *Arabidopsis thaliana* genotypic diversity and *Hyaloperonospora arabidopsidis (Hpa)* on rosette diameter in a pair-wise interaction experiment. A linear mixed model was used to analyse each factor and all interactions between them. Fixed effects included experimental repeat, genotype, cultivation (2-way mixture/monoculture) and *Hpa* (presence/absence). Non-significant terms were eliminated from the model. *F* and *P* values refer to ANOVA tests of each factor separately and the interactions between them. N=1600.

| **Fixed term** | **F** | **n.d.f.** | **d.d.f.** | **P** |
|---|---|---|---|---|
| Experiment | 2145.41 | 1 | 584.9 | <0.001 |
| Genotype | 174.29 | 3 | 584.9 | <0.001 |
| Cultivation | 0.08 | 1 | 584.9 | 0.771 |
| *Hpa* | 334.82 | 1 | 584.9 | <0.001 |
| Experiment. Genotype | 12.57 | 3 | 584.9 | <0.001 |
| Experiment. *Hpa* | 146.6 | 1 | 584.9 | <0.001 |
| Genotype. *Hpa* | 100.35 | 3 | 584.9 | <0.001 |
| Experiment. Genotype. *Hpa* | 13.73 | 3 | 584.9 | <0.001 |
