# Supplementary material for: Impact of disease on diversity and productivity of plant populations
Source: Funct Ecol. 2015 Sep 23;30(4):649–57. doi: 10.1111/1365-2435.12552 (PMC4974914; doi:10.1111/1365-2435.12552)
Supplement: Supplementary file 13 — Table S5 Results from linear mixed modelling to evaluate the effect of Arabidopsis thaliana genotypic diversity and Hyaloperonospora arabidopsidis (Hpa) on days to flower in a pair‐wise interaction experiment. [file FEC-30-649-s013.pdf]

Table S5. The effect of *Arabidopsis thaliana* genotypic diversity and *Hyaloperonospora arabidopsidis (Hpa)* on days to flower in a pair-wise interaction experiment. A linear mixed model was used to analyse each factor and all interactions between them. Fixed effects included experimental repeat, genotype, cultivation (2-way mixture/monoculture) and *Hpa* (presence/absence). Non-significant terms were eliminated from the model. *F* and *P* values refer to ANOVA tests of each factor separately and the interactions between them. N=1600.
.

**Fixed term**	**F**	**n.d.f.**	**d.d.f.**	**P**
Experiment	3265.15	1	446.3	<0.001
Genotype	57.10	3	434.7	<0.001
Cultivation	0.008	1	429.3	0.771
Hpa	2.49	1	426.0	0.115
Experiment. Genotype	11.89	3	544.4	<0.001
Experiment. *Hpa*	39.83	1	443.5	<0.001
Genotype. *Hpa*	8.83	3	433.7	<0.001
Experiment. Genotype. Cultivation	3.61	7	479.6	<0.001
Experiment. Genotype. *Hpa*	11.72	3	551.8	<0.001
Experiment. Genotype. Cultivation. *Hpa*	4.94	7	449.2	<0.001
