# Supplementary material for: Impact of disease on diversity and productivity of plant populations
Source: Funct Ecol. 2015 Sep 23;30(4):649–57. doi: 10.1111/1365-2435.12552 (PMC4974914; doi:10.1111/1365-2435.12552)
Supplement: Supplementary file 14 — Table S6 Results from linear mixed modelling to evaluate the effect of Arabidopsis thaliana genotypic diversity and Hyaloperonospora arabidopsidis (Hpa) on seed productivity in a competitive interaction experiment. [file FEC-30-649-s014.pdf]

**Table S6.** The effect of *Arabidopsis thaliana* genotypic diversity and *Hyaloperonospora arabidopsidis (Hpa)* on seed productivity in a pair-wise interaction experiment. A linear mixed model was used to analyse each factor and all interactions between them. Fixed effects included experimental repeat, genotype, cultivation (monoculture,/2-way mixture,/4-way mixture), *Hpa* (presence/absence) and cultivation (mixture/monoculture). Non-significant terms were eliminated from the model. *F* and *P* values refer to ANOVA tests of each factor separately and the interactions between them. N=1600.

| **Fixed term** | **F** | **n.d.f.** | **d.d.f.** | **P** |
|---|---|---|---|---|
| Experiment | 236.07 | 1 | 636.1 | <0.001 |
| Genotype | 1.65 | 3 | 1116.6 | 0.2 |
| Cultivation | 6.76 | 2 | 484.5 | 0.001 |
| *Hpa* | 0 | 1 | 635.0 | 0.98 |
| Experiment. Genotype | 130.56 | 3 | 1111.8 | <0.001 |
| Genotype. Cultivation | 6.44 | 6 | 1179.4 | <0.001 |
| Experiment. *Hpa* | 60.04 | 1 | 636.6 | <0.001 |
| Genotype. *Hpa* | 48.26 | 3 | 1112.9 | <0.001 |
| Experiment. Genotype. *Hpa* | 10.09 | 3 | 1110.4 | <0.001 |
| Genotype. Cultivation. *Hpa* | 2.44 | 8 | 990.4 | 0.01 |
