# Supplementary material for: Impact of disease on diversity and productivity of plant populations
Source: Funct Ecol. 2015 Sep 23;30(4):649–57. doi: 10.1111/1365-2435.12552 (PMC4974914; doi:10.1111/1365-2435.12552)
Supplement: Supplementary file 15 — Table S7 Results from linear mixed modelling to evaluate the effect of Arabidopsis thaliana genotypic diversity and Turnip yellows virus (TuYV) on days to flower in a pair‐wise interaction experiment. [file FEC-30-649-s015.pdf]

Table S7. The effect of *Arabidopsis thaliana* genotypic diversity and *Turnip yellows virus* (TuYV) on days to flower in a pair-wise interaction experiment. Fixed effects included genotype, TuYV (presence/absence) and cultivation (mixture/monoculture). Non-significant terms were eliminated from the model. *F* and *P* values refer to ANOVA tests of each factor separately and the interactions between them. N=400.

**Fixed term**	**F**	**n.d.f.**	**d.d.f.**	**P**
Genotype	442.18	1	165.7	<0.001
TuYV	3.55	1	164.4	0.06
Cultivation	9.52	1	164.6	0.002
Genotype. TuYV	7.80	1	166.3	0.006
TuYV. Cultivation	19.73	1	164.9	<0.001
Genotype. TuYV. Cultivation	4.12	2	166.5	0.02
