# Supplementary material for: Impact of disease on diversity and productivity of plant populations
Source: Funct Ecol. 2015 Sep 23;30(4):649–57. doi: 10.1111/1365-2435.12552 (PMC4974914; doi:10.1111/1365-2435.12552)
Supplement: Supplementary file 16 — Table S8 Results from linear mixed modelling to evaluate the effect of Arabidopsis thaliana genotypic diversity and Turnip yellows virus (TuYV) on rosette diameter in a pair‐wise interaction experiment. [file FEC-30-649-s016.pdf]

Table S8. The effect of *Arabidopsis thaliana* genotypic diversity and *Turnip yellows virus* (TuYV) on rosette diameter in a pair-wise interaction experiment. Fixed effects included genotype, (TuYV) (presence/absence) and cultivation (mixture/monoculture). Non-significant terms were eliminated from the model. *F* and *P* values refer to ANOVA tests of each factor separately and the interactions between them. N=400.

**Fixed term**	**F**	**n.d.f.**	**d.d.f.**	**P**
Genotype	90.99	1	165.0	<0.001
TuYV	3.5	1	165.0	0.06
Cultivation	2.24	1	165.0	0.1
Genotype. Cultivation	3.96	1	165.0	0.05
