# Supplementary material for: Impact of disease on diversity and productivity of plant populations
Source: Funct Ecol. 2015 Sep 23;30(4):649–57. doi: 10.1111/1365-2435.12552 (PMC4974914; doi:10.1111/1365-2435.12552)
Supplement: Supplementary file 17 — Table S9 Results from linear mixed modelling to evaluate the effect of Arabidopsis thaliana genotypic diversity and Turnip yellows virus (TuYV) on seed productivity in a pair‐wise interaction experiment. [file FEC-30-649-s017.pdf]

Table S9. The effect of *Arabidopsis thaliana* genotypic diversity and *Turnip yellows virus* (TuYV) on seed productivity in a pair-wise interaction experiment. A linear mixed model was used to analyse each factor and all interactions between them. Fixed effects included genotype, TuYV (presence/absence) and cultivation (mixture/monoculture). Non-significant terms were eliminated from the model. *F* and *P* values refer to ANOVA tests of each factor separately and the interactions between them. N=400.

**Fixed term**	**F**	**n.d.f.**	**d.d.f.**	**P**
Genotype	18.22	1	137.6	<0.001
TuYV	72.51	1	138.0	<0.001
Cultivation	0	1	137.8	0.9
Genotype. TuYV	56.56	1	137.7	<0.001
Genotype. Cultivation	19.22	1	137.6	<0.001
Genotype. TuYV. Cultivation	6.58	2	138.2	0.002
